# Atherosclerotic cardiovascular disease and dermatomyositis: an analysis of the Nationwide Inpatient Sample survey

**DOI:** 10.1186/ar4135

**Published:** 2013-01-08

**Authors:** Eleni Linos, David Fiorentino, Bharathi Lingala, Eswar Krishnan, Lorinda Chung

**Affiliations:** 1 Department of Dermatology, University of California San Francisco, 2340 Sutter Street, San Francisco, CA 94143, USA; 2 Department of Dermatology, Division of Rheumatology, Stanford University School of Medicine, 291 Campus Drive, Stanford, CA 94305-5101, USA; 3 Department of Medicine, Division of Rheumatology, Stanford University School of Medicine, 291 Campus Drive, Stanford, CA 94305-5101, USA; 4 Palo Alto VA Health Care System, 3801 Miranda Avenue, Palo Alto, CA 94304, USA

## Abstract

**Introduction:**

Increased rates of cardiovascular disease are implicated in several rheumatologic diseases. Our aim was to characterize dermatomyositis hospitalizations and evaluate cardiovascular-associated mortality in this patient population.

**Methods:**

We examined the frequency and mortality rates of several atherosclerotic cardiovascular diagnoses and procedures among hospitalized adult patients with dermatomyositis using data from the US Nationwide Inpatient Sample (NIS) from 1993 to 2007. We compared the odds of death among hospitalized dermatomyositis patients with each cardiovascular diagnosis or procedure to those without, as well as to controls with cardiovascular diagnoses, using logistic regression.

**Results:**

A total of 50,322 hospitalizations of dermatomyositis patients occurred between 1993 and 2007 (mean age 58 years, and 73% female). Of all dermatomyositis hospitalizations, 20% were associated with a concurrent atherosclerotic cardiovascular diagnosis or procedure. The overall in-hospital mortality was 5.7%. Dermatomyositis patients with any associated atherosclerotic cardiovascular diagnosis or procedure were twice as likely to die during the inpatient stay compared to dermatomyositis patients who did not have atherosclerotic cardiovascular disease (OR = 2.0 95% CI 1.7-2.5, p < 0.0001). The odds ratio for death in patients with both dermatomyositis and cardiovascular disease compared to controls with cardiovascular disease alone was 1.98 (95% CI 1.57-2.48) in multivariate adjusted models.

**Conclusions:**

Approximately one fifth of dermatomyositis hospitalizations in the US were associated with an atherosclerotic cardiovascular diagnosis or procedure. These patients have double the risk of in-hospital death in comparison with controls and dermatomyositis patients without a cardiovascular diagnosis, making identification of these groups important for both prognostic purposes and clinical care.

## Introduction

Ischemic heart disease is more prevalent in rheumatologic diseases such as systemic lupus erythematosus and rheumatoid arthritis [1-3] than in the general population. Accelerated atherosclerosis in these populations is thought to be related to the associated inflammatory state, increased prevalence of cardiovascular risk factors, and side effects of therapies such as corticosteroids [4]. Dermatomyositis is a rare autoimmune disease characterized by symmetric proximal muscle weakness and an associated cutaneous eruption. To date, it is not clear whether atherosclerotic cardiovascular disease (CVD) is more prevalent in patients with dermatomyositis compared with the general population. Cardiac manifestations in patients with dermatomyositis include congestive heart failure, arrhythmias, myocardial fibrosis, and myocardial infarction (MI) [5]. Prior studies have estimated that approximately 15% to 55% of deaths in patients with dermatomyositis are due to CVD [6-8]. A recent study in a South Australian population showed that the prevalence of atherosclerotic risk factors, including hypertension and diabetes, is significantly higher in patients with inflammatory myopathies [9] than in the general population. This Australian study included only 43 patients with dermatomyositis and had missing data on more than half of these patients. In a Canadian cohort, the incidence of acute MI was found to be almost twice as high in patients with dermatomyositis or polymyositis compared with age- and gender-matched controls without an inflammatory myositis, but there was no estimation of prevalence or associated mortality risk [10].

The burden of CVD in hospitalized patients with dermatomyositis has not been described to date. The aims of this analysis were to describe dermatomyositis hospitalizations in the US and to assess cardiovascular-related mortality in a representative sample of US inpatients.

## Materials and methods

### Data

This is a case-control analysis of the Healthcare Cost and Utilization Project Nationwide Inpatient Sample (NIS) for the period of 1993 to 2007. The NIS is composed of a stratified sample from 20% of US community hospitals and contains about 7 million hospital discharge records per year. Data from discharge summaries are abstracted into the database for each individual hospitalization. The variables collected for each hospitalization are age, gender, type of hospitalization (elective versus non-elective), discharge diagnosis (primary and up to 14 secondary), procedures performed during the hospitalization, and in-hospital mortality. As we used data that are available to the public, the institutional review board at Stanford University considered the study exempt from review.

### Inclusion and exclusion criteria

We identified all hospitalizations for patients who were at least 18 years old and who had an International Classification of Disease-9 Clinical Modification (ICD-9) for dermatomyositis (710.3) as either the primary or any of the secondary diagnoses. To increase the specificity for a diagnosis of dermatomyositis, we excluded hospitalizations in which there was a concurrent rheumatologic diagnosis of polymyositis (710.4), systemic sclerosis (701.1), systemic lupus erythematosus (710.0), or rheumatoid arthritis (714.0). We also excluded hospitalizations for childbirth.

### Validation of ICD-9 codes

In a previous study by our group, the ICD-9 code of 710.3 was validated against detailed medical record review and was found to be adequately valid for a probable diagnosis of dermatomyositis according to Bohan and Peter criteria with a positive predictive value of 70% [11].

### Atherosclerotic cardiovascular disease

Among this group, those with a concomitant discharge diagnosis of acute or chronic CVDs or cardiovascular procedures were identified. These included coronary artery disease and acute MI (410.0 to 410.9, 412), unstable angina (411.1, 411.81, 411.89), angina pectoris (413, 413.1, 413.9), cardiac catheterization (372.1 to 372.3), percutaneous transluminal coronary angioplasty (360.1 to 360.9), coronary artery bypass grafting (361.0 to 361.9), and congestive heart failure (428.0 to 428.9).

### Comparison group

The control group consisted of age- and gender-matched hospitalizations from the general discharge population (10 per case) during the same time period as dermatomyositis admissions. The control group did not include diagnoses of rheumatologic diseases specified in the exclusion criteria.

### Statistical analyses

We compared the prevalence of cardiovascular diagnoses or procedures as well as in-hospital mortality between patients with dermatomyositis and controls. All analyses were weighted to account for the survey sampling procedure and to produce representative national estimates. We developed univariate and multivariate logistic regression models to estimate the odds of in-hospital mortality by comparing dermatomyositis patients with CVD to controls with CVD as well as dermatomyositis patients with or without CVD.

### Covariates

Covariates for multivariate analysis included important clinical risk factors such as age, gender, type of admission (elective versus non-elective), and the Charlson comorbidity index [11], which reflects 1-year mortality due to comorbidities (scale of 0 to 37), to account for concomitant illnesses [12]. The Charlson index is a prediction index that includes the following diagnoses: MI, congestive heart failure, peripheral vascular disease, dementia, chronic pulmonary disease, connective tissue disease, ulcer disease, mild liver disease, diabetes, renal disease, non-metastatic solid tumor, leukemia, lymphoma, myeloma, metastatic tumor, or AIDS. We also adjusted for the number of concurrent CVD diagnoses in order to control for possible differences in reporting accuracy. All analyses were performed by using the SVY suite of SAS 9 (SAS Inc., Cary, NC, USA).

## Results

### Description of the study sample

A total of 10,156 hospitalizations were recorded in the database, representing 50,323 hospitalizations of patients with dermatomyositis in the US between 1993 and 2007. Baseline characteristics are shown in Table 1. Half of these admissions occurred in teaching hospitals, 39% in urban hospitals, and 11% in rural hospitals. The mean age of patients was 58 years, and 73% of patients were female. Approximately half of all hospitalized patients with dermatomyositis were white, 12% were black, 8% were Hispanic, and 4% were other. The mean length of hospitalization was 8 days. The mean Charlson comorbidity index in our overall dermatomyositis patient group was 1.4.

**Table 1:** Characteristics of patients and controls hospitalized for dermatomyositis (1993 to 2007)

Characteristics	Patients with dermatomyositis	Controls^a^
Total number in sample	10,156	76,440
Weighted number (to US population)	50,323	376,744
Mean age, years	58.3	58.5
Percentage of females	73.2	73.4
Race, percentage		
White	53.2	71.4
Black	11.8	15.5
Hispanic	7.6	10.1
Other	3.7	3.0
Missing	23.6	0
Income, percentage		
1 (lowest category)	19.7	28.2
2	22.4	23.8
3	23.7	22.3
4 (highest category)	31.3	22.8
Missing	2.9	2.9
Hospital type		
Rural	11.6	11.5
Urban, non-teaching	39.3	43.5
Urban, teaching	49.1	45.0
Mean length of hospitalization, days	8.0	5.1
Mean total charges, US dollars	28,545	33,853
Any cardiovascular diagnosis, percentage	20.4	21.1
MI or acute coronary artery disease	4.4	5.7
Angina	1.2	1.7
Angina pectoris	1.2	1.3
Angina (either)	2.5	2.8
Congestive heart failure	11.8	9.9
Coronary artery bypass graft	0.4	0.01
Coronary catheterization	2.9	0
Percutaneous transluminal coronary angioplasty	0.8	0
Charlson comorbidity index	1.4	1.2
Overall in-hospital death rate per 1,000	56.5	24.0

^a^Controls were matched for age and gender. MI, myocardial infarction.

The most frequent primary discharge diagnoses in hospitalizations of patients with dermatomyositis were pneumonia and acute respiratory failure, congestive heart failure, coronary atherosclerosis, sepsis, cellulitis, and urinary tract infection. Of all dermatomyositis hospitalizations, one fifth were associated with a concurrent atherosclerotic cardiovascular diagnosis or procedure, which was similar to the control group (Table 1). The most common cardiovascular diagnosis in this group of patients was congestive heart failure (12% of dermatomyositis hospitalizations) followed by MI (4.4% of dermatomyositis hospitalizations).

The overall in-hospital mortality in our dermatomyositis patient group was 5.7% over the study period. The most frequent primary diagnoses in dermatomyositis patients who died in the hospital were pneumonia (including aspiration pneumonia), acute respiratory failure, sepsis, and congestive heart failure. Dermatomyositis patients with any of the specified atherosclerotic cardiovascular diagnoses or procedures were twice as likely to die during the inpatient stay compared with dermatomyositis patients who did not have a concurrent atherosclerotic cardiovascular diagnosis or procedure (odds ratio (OR) = 2.0, 95% confidence interval (CI) 1.7 to 2.5, *P *< 0.0001) (Table 2). The highest odds of death were noted for patients with congestive heart failure (OR = 2.3, 95% CI 1.9 to 2.8, *P *< 0.001). The odds of death were about twofold higher for hospitalized patients with dermatomyositis and CVD compared with hospitalized age- and gender-matched control patients with CVD, and this finding persisted after adjustment for comorbidities, admission type, and number of cardiovascular diagnoses/procedures (OR = 1.98, 95% CI 1.57 to 2.48) (Table 3).

**Table 2:** Odds ratio for death, comparing patients with dermatomyositis and cardiovascular disease diagnoses with dermatomyositis patients without cardiovascular disease

Cardiovascular diagnoses	Odds ratio (95% CI)	*P *value
Any cardiovascular diagnosis	2.04 (1.71-2.45)	< 0.001
Myocardial infarction	1.57 (1.11-2.22)	0.01
Angina	0.34 (0.14-0.83)	0.02
Congestive heart failure	2.28 (1.85-2.80)	< 0.001
Coronary artery bypass grafting	-	-
Coronary catheterization	0.51 (0.26-1.01)	0.053
Percutaneous angioplasty	0.47 (0.12-1.94)	0.30

CI, confidence interval.

**Table 3:** Odds ratio for in-hospital death, comparing patients with dermatomyositis and cardiovascular disease with controls with cardiovascular disease

Model	Odds ratio (95% CI)	*P *value
Univariate model^a^	2.11 (1.80-2.48)	< 0.001
Multivariate model^b^	1.98 (1.57-2.48)	< 0.001

^a^Adjusted for age and gender only; ^b^adjusted for age, gender, Charlson comorbidity index, number of diagnoses (to control for severity of admission), and type of admission (elective versus emergency). CI, confidence interval.

## Discussion

In this large US-based hospitalization database, approximately one fifth of hospitalizations in patients with dermatomyositis were associated with an atherosclerotic cardiovascular diagnosis or procedure. Congestive heart failure and coronary atherosclerosis were the second and third most frequent primary diagnoses associated with dermatomyositis hospitalizations, respectively, and among the 10 most frequent primary diagnoses associated with dermatomyositis patients who died in the hospital. Patients with dermatomyositis and atherosclerotic CVD were almost twice as likely to die in hospital than age- and gender-matched controls with similar CVDs, even after adjustment for comorbidities, emergency admission, and number of cardiovascular diagnoses/procedures. In addition, dermatomyositis patients with CVD were twice as likely to die compared with patients with dermatomyositis and no CVD. This suggests an additive effect of CVD and dermatomyositis on risk of death. In addition to helping clinicians with inpatient prognostication, this finding raises important clinical questions about the possible need for more aggressive cardiovascular risk factor assessment and monitoring in dermatomyositis outpatients in order to prevent hospitalization and death related to CVD.

Inflammatory myopathies have been linked to cardiovascular risk factors, including diabetes, hypertension, and ischemic heart disease. In a recent study, the prevalence of hypertension and diabetes in these patients was far higher than in the general population (62% and 29% compared with 9% and 4%, respectively) [9]. Another study found that the incidence of acute MI and cerebrovascular accident was higher in patients with dermatomyositis and polymyositis than in age- and gender-matched controls. In that study, the presence of hypertension and hyperlipidemia increased the risk of arterial events whereas the use of non-steroid immunomodulators decreased the risk [10]. Although we were not able to examine detailed risk factor profiles in individual patients in this study, it is possible that hypertension and diabetes may be appropriate targets for screening and early intervention in patients with dermatomyositis. Future studies evaluating the potential benefit of more intense glucose and blood pressure monitoring in the outpatient setting in this population would be helpful. The risk of statins in patients with inflammatory myopathies may complicate the treatment of hyperlipidemia; however, screening and treatment with lifestyle changes and non-statin medications may also be warranted in this population.

The increased mortality observed in patients with both dermatomyositis and atherosclerotic CVD compared with inpatients with only one of those diagnoses is significant for clinical prognostication. Our findings are consistent with those of a prior retrospective cohort study of 370 patients with inflammatory myositis (dermatomyositis and polymyositis) indicating that CVD accounts for most of the excess mortality seen in these patients. In that study, the relative risk of death comparing patients with ischemic heart disease to those without was 2.97 (95% CI 1.65 to 5.28) [13]. Our findings are also consistent with those of other studies showing that cardiovascular involvement contributes to a significant proportion of mortality in patients with dermatomyositis [14,15].

Strengths of this study include the very large numbers of cases included for such a rare disease as well as the specific focus on patients with dermatomyositis. The pathophysiology of dermatomyositis likely differs from that of other inflammatory myopathies, such as polymyositis and inclusion body myositis, as illustrated by differences in clinical expression, muscle biopsy findings, and gene expression analyses. Therefore, dermatomyositis may represent a subgroup of inflammatory myopathies with a particular risk associated with atherosclerotic CVD.

Limitations of this study include the cross-sectional design, which cannot assess temporal relationships between dermatomyositis and cardiovascular diagnoses. Furthermore, this study relied on ICD-9 codes for the definition of dermatomyositis, and this could have led to misclassification. However, we recently found that the ICD-9 code of 710.3 was 70% accurate in identifying cases of dermatomyositis that fulfill Bohan and Peter criteria for at least probable disease [11]. We also attempted to reduce this misclassification by excluding patients who had an additional concurrent rheumatologic diagnosis. Because the unit of analysis was hospital discharge and not the individual patient, it is possible that some patients would have been included more than once if they had multiple hospitalizations. Because ethnicity is not reported by all states, we did not adjust our analyses for race since the proportion of missing data for this covariate was high (23%). The major limitation of this study is that this is an administrative database and so it is not possible to evaluate all possible variables. For example, this dataset does not have information on smoking status, medications, or laboratory measures of inflammation that would inform potential mechanisms. Finally, since our study used a hospitalization database, these results may not be generalizable to all dermatomyositis patients who are typically managed in the outpatient setting.

## Conclusions

One fifth of all hospitalizations for dermatomyositis have an associated atherosclerotic cardiovascular diagnosis or procedure. Patients with both dermatomyositis and CVD have double the risk of in-hospital death compared with hospitalized age- and gender-matched controls with CVD and compared with hospitalized dermatomyositis patients without CVD. Identification of these groups is important for both prognostic purposes and clinical care. Further prospective studies are necessary to confirm our findings and to analyze the relative contribution of disease activity and treatments on the increased mortality associated with CVD in patients with dermatomyositis. Future studies are also needed to assess whether systematic outpatient screening for and treatment of cardiovascular risk factors, including diabetes, hyperlipidemia, or hypertension, may be beneficial in reducing cardiovascular mortality in patients with dermatomyositis.

## Abbreviations

CABG: coronary artery bypass grafting; CI: confidence interval; CVD: cardiovascular disease; ICD-9: International Classification of Disease-9 Clinical Modification; MI: myocardial infarction; NIS: Nationwide Inpatient Sample; OR: odds ratio.

## Competing interests

The authors declare that they have no competing interests.

## Authors' contributions

EL, DF, and LC helped to conceive and design the study, had access to data, and helped to draft the manuscript. BL carried out the analysis. EK helped to draft the manuscript. All authors read and approved the final manuscript.
